# Medication error rates in Iranian hospitals: a meta-analysis

**DOI:** 10.1186/s12913-024-11187-6

**Published:** 2024-06-17

**Authors:** Parvaneh Isfahani, Aliyeh Bazi, Samira Alirezaei, Somayeh Samani, Mohammad Sarani, Fatemeh Boulagh, Mahdieh Poodineh Moghadam, Mahnaz Afshari

**Affiliations:** 1 Department of Health Management, School of Public Health, Zabol University of Medical Sciences, Zabol, Iran; 2 Department of Clinical Pharmacy, School of Pharmacy, Zabol University of Medical Sciences, Zabol, Iran; 3 Research Center for Social Determinants of Health, Saveh University of Medical Sciences, Saveh, Iran; 4 Department of Occupational Health Engineering, School of Public Health, Zabol University of Medical Sciences, Zabol, Iran; 5 Department of Public Health, School of Public Health, Zabol University of Medical Sciences, Zabol, Iran; 6 Department of Nursing, School of Nursing and Midwifery, Zabol University of Medical Sciences, Zabol, Iran

**Keywords:** Medication Error, Patient, Hospital, Meta-analysis

## Abstract

**Background and aim:**

Medication errors (MEs) in hospitals decrease patient satisfaction, increase hospital mortality, lower hospital productivity, and increase in the costs of the health system. This study was conducted to determine the rate of MEs in Iranian hospitals.

**Method:**

In this meta-analysis, all published articles on ME rates in Iranian hospitals were identified from five databases and Google Scholar and assessed for quality. The heterogeneity of the studies was examined using the I^2^ index and a meta-regression model was used to evaluate the variables suspected of heterogeneity at the 0.05 significance level. Finally, 17 articles were eligible to be included in this study and were analyzed using the Comprehensive Meta‐Analysis (CMA) software.

**Findings:**

Based on the estimation of the random-effects model, the ME rate in Iranian hospitals was 10.9% (5.1%-21.7%; 95% CI). The highest rate was observed in Sanandaj in 2006 at 99.5% (92.6%-100.0%; 95% CI) and the lowest rate was observed in Kashan in 2019 at 0.2% (0.1%-0.3%; 95% CI). In addition, sample size and publication year were significantly correlated with ME rate (*P* < 0.05).

**Conclusion:**

According to the results of this study; ME rate in Iran is relatvively high based on the synthesis of the research conducted in Iranian hospitals. In addition to being costly, MEs have negative consequences for patients. Thereofore, it is necessary to emphasize the voluntary nature of medication error reporting in health sytem of Iran.

## Introduction

Hospitals are an integral part of the medical and social systems, responsible for providing health and medical care to all people in all communities. Patients are looking for quality, safe, effective, and efficient diagnostic and therapeutic services. Therefore, one of the most important goals of hospitals is to provide quality, safe, and effective care to patients and meet their reasonable needs and expectations [1, 2]. However, this is a very complex process that involves different people and equipment. The complex nature of these services can increase the likelihood of medication errors (MEs) in hospital settings [3].

MEs are a major problem in every health system worldwide, causing physical complications and death in patients [4]. MEs are one of the five categories of medical errors as defined by the American Medical Association [5]. They can occur at any stage of patient care delivery and cause serious adverse effects. This type of medical error threatens the health and well-being of patients, and its recurrence can undermine the quality of care [6]. ME is a failure in the treatment process that leads to possible harm to patients [7]. The first reports of MEs occurred in 1940 and drew the attention of many researchers [8].

The National Coordinating Council on Medication Error Reporting and Prevention (NCC MERP) defines medication error as “any preventable event that may cause or lead to inappropriate medication use or patient harm while the medication is in the control of the health care professional, patient, or consumer” [9]. MEs may occur at any stage of medication use process, including prescribing, documenting, transcribing, dispensing, administering, and/or monitoring. In the prescription phase, the most common type of error is writing the wrong medicine, the wrong dose, and/or the wrong frequency by the health care professional [10]. Prescription errors account for approximately 50% of MEs. In general, MEs are a pervasive problem, but in most cases they are preventable [11].

According to the World Health Organization (WHO), various factors could lead to MEs, including factors associated with: health care providers (lack of training, inadequate knowledge and experience, inadequate knowledge of the patient, inadequate risk perception, overwork and fatigue, physical and emotional problems, poor communication with the patient); the patient (patient characteristics and the complexity of the disease); the work environment (pressure, distractions and interruption in the work process, lack of protocols and clinical guidelines, insufficient resources, shortage of safety equipment); drugs (name of drugs, drug packaging and labeling); tasks (patient monitoring, repetitive processes); computerized information systems (inadequate design, difficult processes for generating information, inaccurate patient records); and the primary-secondary care interface (limited communication with secondary care, poorly justified secondary care recommendations) [12]. Therefore, a combination of human and organizational factors cause MEs.

MEs and their adverse effects can lead to longer hospital stays, increased medical expenses, disability, and death [5, 6]. Some studies have shown that MEs account for approximately 7,000 deaths in every 44,000 to 98,000 deaths that occur due to medical errors [13, 14]. In addition, such errors result in dissatisfaction and undermine patients’ trust in the health system [5, 6]. In the United States, about 7,000 to 9,000 people die each year due to MEs. Moreover, hundreds of thousands of people experience MEs but do not report adverse reactions or complications. The total cost of caring for patients with MEs is over $40 billion per year. In addition to higher medical expenses, patients experience a lot of mental and physical trauma as a result of MEs [15, 16].

Several studies have examined ME rates in Iranian hospitals, but reported rates have been variable [17–19]. For example, a 2008 study in Tehran showed that the average rate of MEs within a span of three months was about 19.5 cases for each nurse [17]. Another study in Kermanshah showed that ME rate among nurses in 2013 was about 79.2% [18]. ME and its adverse effects are inevitable in any health system. Therefore, trying to improve patient safety and reduce MEs is a challenging task that requires accurate clinical and non-clinical processes as well as adequate resources. The principles and techniques of risk management help hospital managers prevent the substantial human and financial harm caused by MEs. Therefore, the purpose of the present study was to measure the rate of MEs in Iranian hospitals.

## Method

The literature search for this systematic review was adopted based on the Preferred Reporting items for Systematic Reviews and Meta-Analyses checklist (PRISMA) guidelines [20].

### Search strategy

The relevant evidence was extracted from several English and Persian databases, including Web of science, Scopus, PubMed, SID, Magiran, and Google Scholar using the following keywords and their Persian equivalents: Medication error, frequency, hospital, and Iran. The reference lists of previous studies were examined for further relevant articles, and keywords were combined with Boolean operators, including AND and OR. The search strategy is provided in Table 1. The extracted articles were reviewed in EndNote X9.

**Table 1:** List of terms used and search results

Database	Scopus	Web of Science	PubMed	Magiran	SID	Googlescholare
Search Sterategy	ALL ( "medication error") AND ALL ( frequency) AND ALL ( hospital) AND ALL ( iran) AND ( LIMIT-TO ( DOCTYPE, "ar")) AND ( LIMIT-TO ( LANGUAGE, "English") OR LIMIT-TO ( LANGUAGE, "Persian")) AND ( LIMIT-TO ( PUBSTAGE, "final")) AND ( LIMIT-TO ( OA, "all"))	(((ALL = ("medication error")) AND ALL = (hospital)) AND ALL = (frequency)) AND ALL = (Iran) and Open Access and Article (Document Types) and All Open Access (Open Access)	("medication error"[All Fields] AND ("hospital s"[All Fields] OR "hospitalisation"[All Fields] OR "hospitalization"[MeSH Terms] OR "hospitalization"[All Fields] OR "hospitalised"[All Fields] OR "hospitalising"[All Fields] OR "hospitality"[All Fields] OR "hospitalisations"[All Fields] OR "hospitalizations"[All Fields] OR "hospitalize"[All Fields] OR "hospitalized"[All Fields] OR "hospitalizing"[All Fields] OR "hospitals"[MeSH Terms] OR "hospitals"[All Fields] OR "hospital"[All Fields]) AND ("epidemiology"[MeSH Subheading] OR "epidemiology"[All Fields] OR "frequency"[All Fields] OR "epidemiology"[MeSH Terms] OR "frequence"[All Fields] OR "frequences"[All Fields] OR "frequencies"[All Fields]) AND ("iran"[MeSH Terms] OR "iran"[All Fields])) AND ((ffrft[Filter]) AND (1000/1/1:2023/9/22[pdat]) AND (english[Filter]))	Medication error AND hospital	Medication error	("medication error") AND hospital AND frequency AND Iran
Total articles	178	6	14	60	40	2160
Search date	Until September 22, 2023	Until September 22, 2023	Until September 22, 2023	Until September 22, 2023	Until September 22, 2023	Until September 22, 2023
Time	September 22, 2023	September 22, 2023	September 22, 2023	September 22, 2023	September 22, 2023	September 22, 2023
language	English	English	English	Persian	Persian	English

### Inclusion criteria of studies

All full text quantitative articles published with English and Persian languages conducted in Iran which reported the rate of medication errors in all ward (Inpatient, Surgery,and Special wards and clinical departments) until September 22, 2023 were entered to the meta-analysis after the evaluation process.

### Exclusion criteria of studies

The articles that did not meet the following criteria were excluded: 1) letter to the editor, case–control, randomized controlled trials and qualitative studies 2) grey literature, books, and dissertations; 3) articles, documents, and reports published after September 22, 2023;4) studies that did not obtain the minimum score of 15; 5) studies published in any language other than English.

### Quality assessment

After determination of the relevant studies in terms of titles and content, the checklist which was.

used in the previous studies was applied to evaluate the quality. The checklist was prepared by examiningthe content of the STROBE list including 22 questions that covered the various aspects of the methodology including determination of the appropriate sample size, research type, sampling, sample data, collection methods, the definition of variables and samples, tools for data collection, statistical analysis, providing results properly, and presenting the results based on the objectives. The distribution of scores for the final checklist according to the number of independent items in each section, in this way, title and abstract 2, introduction 2, methods 10, results 8, discussion 4 and other information score 1 is considered. Therefore studies with at least 15 points [21] were entered to the meta-analysis.

In order to avoid bias, extraction and evaluation of the quality of the article was done by two independent researchers. If the articles are not included, the reason for rejection is mentioned. In cases where there was a difference of opinion between the two researchers, the review of the article was done by a third person.

### Data extraction

Data of each study were extracted due to the title, the first author's name, done year, place of study, the sample size of study, tool, hospital type, quality score, the overall prevalence of medication errors. The data was entered by two researchers in an Excel spreadsheet (Table 2).

**Table 2:** Characteristics of the included studies

No	Author	Year	Place	Total sample	Prevalence (%)	Tool	Causes	Hospital type	Ward	Quality article	Reference
1	Malekzadeh	2014	Mazandaran	1927	24.02	Error report form	Incorrect Drug	Educational	All*	18	[22]
2	Mohammad Alizadeh	2014	Tehran	162	97.53	Error report form	Wrong patient	Educational	All	15	[23]
3	Zaree	2015	Tehran	379	54.08	Questionnaire	Wrong dosage	Public	All	21	[24]
4	Shams	2011	Khoy	350	28.9	Questionnaire	Concomitant administration of oral drugs with potential interaction	Public& private&Educational	All	18	[25]
5	Khammarnia	2012	Shiraz	2951	17.2	Medical error records	Wrong dosage	Public	All	20	[26]
6	Masrour	2012	Tehran	200	38.5	Questionnaire	Drug omission	Educational	All	19	[27]
7	Mirzaei	2012	Kermanshah	96	79.2	Questionnaire	Incorrect Drug	Educational	All	22	[18]
8	Hashemian	2019	Hamadan	903	42.6	Error report form	Wrong dosage	Educational	All	16	[28]
9	Baghaei	2015	Urmia	84	42.9	Questionnaire	Forgetting to give medication	Educational	All	18	[29]
10	Fathi	2016	Kermanshah	500	17	Questionnaire	Incorrect time	Educational	All	19	[30]
11	Yousefi	2019	Kashan	6020	9.45	Questionnaire	Incorrect time	Public	All	22	[31]
12	Joolaee	2009	Tehran	286	19.5	Questionnaire	Incorrect time	Educational	All	18	[32]
13	Fahimi	2009	Tehran	558	29.9	Direct obsevarion	Drug omission	Educational	All	20	[33]
14	Davoodi	2013	Mashhad	1000	46	Questionnaire	Incorrect Drug	Educational	All	20	[34]
15	Zahmatkeshan	2006	Bushehr	400	49.7	Questionnaire	Wrong dosage	Educational	All	20	[35]
16	Penjvini	2006	Sanandaj	104	16.7	Questionnaire	Wrong dosage	Educational	All	18	[36]
17	Ahangarzadeh	2010	Sanandaj	100	100	Questionnaire	Wrong patient	Educational	All	20	[37]

*Inpatient, Surgery,and Special wards and clinical departments

### Analysis

Data was exchanged to the Comprehensive Meta-Analysis program (Adaptation 2.2.064) for analysis. Heterogeneity between studies was determined utilizing Cochran's Q-test and I^2^ index. The I^2^ Index was 98.61%, demonstrating the heterogeneity of the studies. Hence, a random-effects model was utilized in this meta-analysis. The effect of variables that may be the potential sources of heterogeneity was examined utilizing the met regression technique. At last, by utilizing the met regression function, the effect of variables, which potentially accounted for the heterogeneity within the included studies, was examined. The point estimate of the prevalence of medication errors was calculated at the 95% confidence interval (CI) in forest plots, where the size of the box shows the weight of each study, and the horizontal line demonstrates the 95% CIs.

## Result

**Fig. 1:**
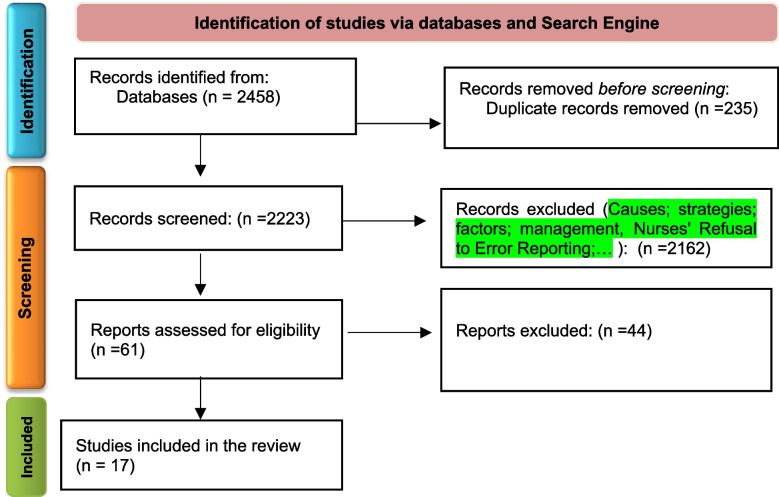
PRISMA flow diagram illustrating study selection process

In total 2458 articles were found by initial search. After removing 235 duplicates, 2223 titles and abstracts were screened and 2162 irrelevant ones were deleted. Finally, 17 articles of 61 reviewed full texts were included in data synthesis (Fig. 1).

**Fig. 2:**
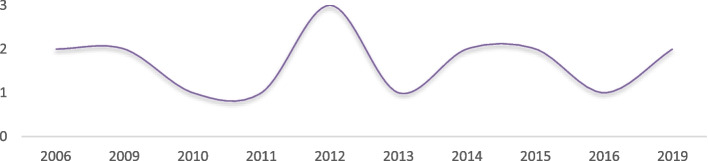
Distribution of reviewed studies by the year of publication

**Fig. 3:**
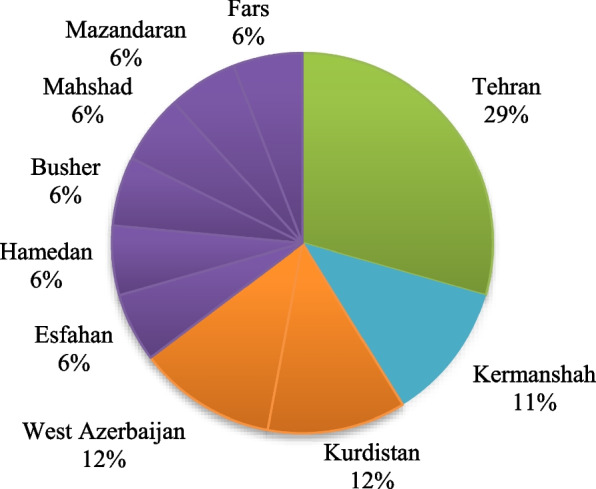
Distribution (%) of reviewed studies by the province

These studies were published more in 2012 (Fig. 2). They were conducted in 10 provinces, mostly in Tehran (5 studies), Kermanshah (2studies), Kurdistan(2 studies) and West Azerbaijan (2 studies) (Fig. 3).

**Fig. 4:**
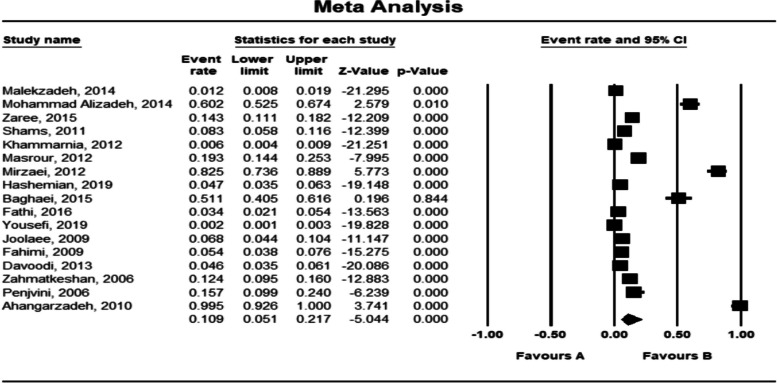
Meta-analysis of the prevalence of medication errors in Iranian hospitals based on the random effects model

Based on the estimation of the random effects model, the prevalence of medication errors in Iranian hospitals was 10.9% (5.1%-21.7%; 95% CI). The highest prevalence rate was observed in Sanandaj in 2006 at 99.5% (92.6%-100.0%; 95% CI) and the lowest prevalence rate was observed in Kashan in 2019 at 0.2% (0.1%-0.3%; 95% CI) (Fig. 4).

The prevalence of medication errors in Iranian hospitals has varied by geographic region, Hospital type, Causes and tool (Table 3). Higher prevalence rates reported in the western provinces (35.8%). The prevalence of medication errors in Iranian hospitals was higher in educational hospitals(17.8%). With questionnaire tool reported higher prevalence (14.8%). The most common medication error was drug omission (%10.4).

**Table 3:** prevalence of medication errors in Iranian hospitals by geographic region, Hospital type and tool

Variables		No. Studies	Prevalence	CI	Heterogeneity	
					Percentage	*P*-value
**Region**	Central	6	8.0	0.2–26.7	98.87	≤ 0.01
	Southern	2	2.8	0.1–3.97	99.19	≤ 0.01
	Northern	3	9.6	0.9–5.45	99.08	≤ 0.01
	Western	5	35.8	7.3–79.7	98.53	≤ 0.01
	Eastern	1	4.6	3.5–6.1	-	-
**Hospital type**	Educational	13	17.8	0.8–34.8	98.51	≤ 0.01
	Public	3	12	0.01–17.9	99.21	≤ 0.01
	Public& private& Educational	1	8.3	5.3–11.6	-	-
**Tool**	Error report form	3	0.9	0.6—6.10	99.50	≤ 0.01
	Questionnaire	12	14.8	6.8–29.4	98.09	
	Medical error records	1	0.6	0.4–0.9	-	-
	Direct obsevarion	1	5.4	0.3–7.6	-	-
**Cuases**	Wrong Patient	2	9.34	1.07–9.99	91.48	≤ 0.01
	Wrong Dosage	5	6.2	2.3–15.9	97.71	≤ 0.01
	Incorrect Drug	3	1.24	0.8–7.22	99.37	≤ 0.01
	Concomitant administration of oral drugs with potential interaction	1	0.8	0.5–1.16	-	-
	Drug omission	2	10.4	2.8–32.2	96.73	≤ 0.01
	Forgetting to give medication	1	5.11	40.5–61.6	-	-
	Incorrect time	3	1.6	0.2–1.13	97.89	≤ 0.01

To examine the factors causing heterogeneity, sample size and done year were included in the meta-regression model. The results are reported in Table 4, indicating that both of these variables have contributed to the heterogeneity of the findings across the studies (*P* < 0.05).

**Table 4:** Results adjusted by the factors causing heterogeneity (the meta-regression model)

Suspected Variables	Correlation Coefficients	P-value
Done Year	-0.05	≤ 0.01
Sample size	− 0.001	≤ 0.01

## Discussion

According to the synthesis of the results of 17 studies conducted so far, the rate of MEs in Iranian hospitals is about 10.9%. On average, 18% of patients in the WHO Eastern Mediterranean Region (consisting of 22 countries including Iran) experience adverse effects [38]. Most of these occur in the areas of medication, treatment, diagnosis, surgery, childbirth, and pediatric care. In the EMRO, each adverse effect leads to 9.1 days longer hospital stays. About 15% of hospital activities and costs are directly spent on adverse effects, costing billions of dollars [39]. It seems that the rate of MEs in Iranian hospitals is consistent with the results reported for EMRO.

In this study, the rate of MEs was found to be higher in the western provinces of Iran. Iran is one of the largest countries in the Middle East region with more than 70 million people of several ethnicities. Azeri people live mostly in the northwest, Kurds in the west, Arabs in the south and southwest, Fars in the center, Turkmen in the northeast, and Baloch in the east of the country. Accumulation of more hospital beds in the center of the province and more developed cities with better living conditions and higher income will lead to more concentration of hospital workers such as doctors and nurses in these cities and It will have a negative effect on people's health (increasing medical errors, unnecessary admissions, hospital infections, etc.) in less privileged cities [40]. Of course, due to the limited number of studies, this finding should be interpreted with caution, and further research in different provinces of the country is needed for effective evaluation and planning.

The reviewed studies used different methods for measuring ME rates, such as error report forms, questionnaires, medical records, and direct observation [33, 27, 26, 22]. The results showed that the ME rate reported using an error report form was 0.9%. Most Iranian hospitals use a voluntary error report form to identify errors, which detects a small number of errors compared to other error screening methods. For instance, a 2014 study in an Iranian training hospital used the Global Trigger tool (accident screening) to screen patient medical records and found that the rate of adverse effects was 1.19 per 100 admissions and 2.57 per 1,000 inpatient days. Approximately 15.9% of patients experienced adverse effects. Meanwhile, the rate of errors identified during one year using a voluntary error reporting form was equal to 0.19%. That is, the accident screening method was 100 times more effective in identifying errors than the voluntary error reporting form [41]. Therefore, the actual rate of MEs in Iranian hospitals is likely to be higher than the numbers reported in this study.

Unfortunately, hospital staff do not report many errors. The main reasons for underreporting of errors in hospitals are the lack of awareness of staff about errors, lack of belief in improving safety by reporting errors, not understanding the error reporting process, fear of the consequences of error reporting, not having time to complete error reporting forms, forgetfulness, lack of support from managers and colleagues, lack of feedback from management, and fear of patient complaints and damage to one’s reputation [4, 42, 43].

Surveying hospital staff, especially nurses, is another method for assessing medical errors in Iran. In this review, the rate of MEs in Iranian hospitals as measured through survey was about 14.8%. It must be noted that this method has lower accuracy due to recall bias [3]. Only one study used direct observation, reporting an ME rate of 5.4% [33]. Wilmer et al. [44] and Flynn et al. [45] showed that direct observation is the best method to assess and diagnose MEs. Additionally, only one study used medical records to collect information, and the reported error rate was 0.6%.

The results also showed that the ME rate decreases by 0.05 per unit increase in publication year. In other words, the time sequence of studies on MEs indicates lower levels of prevalence in recent years compared to previous years. In recent years, important efforts have been made in Iran to increase the quality and improve the safety of health services, which include the establishment of clinical governance, safety-friendly hospitals, and hospital accreditation based on codified standards [46].

In this study, ME rates were higher in Iranian teaching hospitals. Because some public hospitals in the country are teaching hospitals led by faculty with the participation of assistants, their rate of MEs may be higher than other hospitals. On the other hand, the number of people visiting these hospitals is higher due to the low tariffs. In addition to, In this study, the ME rate decreased by 0.001 per unit increase in sample size. In this study, the sample size varies between 84 and 6020. Therefore, it is necessary to ensure that the sample size is representative of the population and to use suitable and accurate sampling techniques.

Based on the results, wrong patient, wrong dosage, incorected drug, concomitant administration of oral drugs with potential interaction, drug omission, Incorrect time, and forgetting to give medication were the reported errors [28–37]. The most common medication error was drug omission. This was similar to study conducted by Fahimi et al. [33] and Lisby et al. who found omitted doses as the most common errors at the dispensing (5/5) and ordering (144/167) stage [22, 47]. A systematic review and meta-analysis from Southeast Asia also reported wrong time, omission error and wrong dosage were the most frequent reported errors [48]. Regarding the incidence of errors drug omitted error was the highest reported errors. This may be due to the working environment/system reasons as supported by a systematic review in 2019 that showed the associations of MEs with systems including: prescribing, order communication, product labeling, packaging, and nomenclature, compounding, dispensing, distribution, administration, education, monitoring, and use [49].

Hospitals are responsible for providing diagnostic, therapeutic, and rehabilitation services, and patients expect to receive quality, safe, and effective hospital services. According to the World Health Organization (WHO), Health services are of high quality if they are effective, safe, patient-centred, and delivered in a timely fashion [50]. Hence, hospital managers must adopt a proactive and preventive approach to MEs. MEs and adverse effects are caused by a combination of human and organizational factors. Hospital managers and staff must have a systematic approach for identifying, evaluating, and controlling errors. They must identify errors, analyze their probability and severity, investigate their causes, and take necessary action to prevent their recurrence.

Hospital managers should develop and promote a safety culture whereby the entire staff is committed to preventing MEs. In addition, promoting a safe work environment, improving work processes, establishing an effective error reporting system, training staff, and increasing the well-being, motivation and satisfaction of staff have a significant role in reducing MEs. Hospital managers must make sure that staff have the necessary knowledge, skills, and competencies to perform their job, and provide them with the necessary specialized training if needed. Unsuitable working conditions such as heat, cold, noise, poor lighting, and lack of resources increase the likelihood of errors. Therefore, managers should create a safe and suitable working environment for staff and provide them with the necessary equipment and supplies. Low levels of well-being and quality of working life among health care professionals can decrease patient safety and increase errors [3].

This article had some limitations as follows: a- these articles were conducted in a small number of provinces in Iran. Therefore, it is recommended that cross-sectional studies be performed in other provinces of Iran as well. b- There was the lack of valuable information (e.g., gender, work shift, number of shifts, work experience, and age) for a detailed survey. It is suggested that researchers include this information in their analysis to be used in systematic reviews and meta-analyses.

## Conclusion

In general, the rate of MEs in Iran seems to be relatively high based on the synthesis of the research conducted in Iranian hospitals. Iranian studies are based on a voluntary error reporting form. Because a low or high percentage cannot be the reason for the occurrence of medication errors in different provinces. Where the rate of error reporting is high, it may be due to the culture of voluntary error reporting, while where the percentage of error reporting is low, this may not be the case. However, this finding should be interpreted with caution given the limited number of studies measuring ME rates in Iranian hospitals and the small sample of patients in those studies. Therefore, it is recommended to conduct further quantitative research throughout the country and complement those with qualitative studies to obtain a more comprehensive picture of ME rates in Iranian hospitals.

## Data Availability

Not applicable.
